# Impact of the COVID-19 Pandemic on Mortality Following Hip and Knee Joint Arthroplasty Surgeries: A Systematic Review and Meta-Analysis

**DOI:** 10.3390/jpm12091441

**Published:** 2022-08-31

**Authors:** Eic Ju Lim, Minboo Kim, Chul-Ho Kim

**Affiliations:** 1 Department of Orthopaedic Surgery, Chungbuk National University Hospital, Chungbuk National University College of Medicine, Cheongju 28644, Korea; 2 Department of Orthopaedic Surgery, Chung-Ang University Hospital, Chung-Ang University College of Medicine, Seoul 06973, Korea

**Keywords:** arthroplasty, COVID-19, SARS-CoV-2, mortality, readmission

## Abstract

We performed a meta-analysis comparing the mortality rates after hip and knee joint arthroplasty between the coronavirus 2019 (COVID-19) pandemic and pre-pandemic periods. The purpose of present study was to investigate the impact of the COVID-19 pandemic on mortality rates after hip and knee joint arthroplasty. We systematically searched the MEDLINE, Embase, and Cochrane Library databases for studies published up to 28 March 2022. We included studies which directly compared mortality rates after hip and knee joint arthroplasty between the COVID-19 pandemic and pre-pandemic periods. The methodological quality of the included studies was assessed using the Methodological Index for Nonrandomized Studies (MINORS). We compared the overall mortality rate as the primary outcome. For the subgroup analysis, the mortality rates included were: within 30 days and unrelated to COVID-19; we excluded studies with only elective arthroplasties. Readmission rates following arthroplasties were also compared. We included five studies with 3044 patients, of which 838 patients underwent surgeries during the pandemic period and 2206 patients underwent surgeries in the pre-pandemic period. The mean MINORS score was 15.4/24 (range: 15–16). The overall mortality rate showed no significant differences between the pandemic and pre-pandemic periods (OR, 2.71; 95% CI, 0.78–9.35; *p* = 0.12; I^2^ = 19%). No differences were observed in mortality following arthroplasties within 30 days and unrelated to COVID-19 nor in the readmission rates. Mortality, after excluding studies with only elective arthroplasty, presented significant differences between the COVID-19 pandemic and pre-pandemic periods (OR, 3.80; 95% CI, 1.18–12.28; *p* = 0.03, I^2^ = 0%). The limitation of the present study was that elective arthroplasty and urgent arthroplasty were not completely differentiated. The overall mortality rate in the COVID-19 pandemic period following hip and knee arthroplasty did not show a significant increase. This finding could help to maintain the practice of elective arthroplasty during a pandemic situation in the future (PROSPERO–CRD42022335471).

## 1. Introduction

The coronavirus disease 2019 (COVID-19) outbreak has affected the orthopedic healthcare system [1]. In the first wave of the COVID-19 pandemic, elective arthroplasties were significantly reduced to provide care for patients with COVID-19 and prevent disease spread [2]. Notably, hospitals have performed urgent surgeries, such as hip fractures or infected arthroplasty with sepsis, according to their guidelines, which have been changed several times due to the waves of the pandemic, as these surgeries cannot be delayed for a long time.

Several studies have been conducted on arthroplasties performed in COVID-19 patients. Forlenza et al. reported that postoperative COVID-19 infection following hip and knee arthroplasty was associated with a higher rate of complications, such as cardiopulmonary compromise, thromboembolic disease, renal injury, and urinary-tract infection [3]. In contrast, Stonehem et al. demonstrated that COVID-19 patients requiring arthroplasty for trauma did not require intensive care unit (ICU) support due to COVID-19 and did not present greater complications than expected [4].

Comparisons between the mortality rates in the COVID-19 pandemic and pre-pandemic periods following hip and knee arthroplasties have also been reported. Agrawal et al. reported no significant increases in mortality following arthroplasty during the pandemic compared with the pre-pandemic period [5]. In contrast, Khanna et al. demonstrated an approximately six-fold increase in complications after arthroplasty during the pandemic period, with high 30-day mortality [6]. However, to the best of our knowledge, no large-scale study has directly compared the mortality rates following arthroplasties during the COVID-19 pandemic and pre-pandemic periods. Therefore, we investigated the mortality rates in the COVID-19 pandemic and pre-pandemic periods with respect to arthroplasty. The purpose of present study was to investigate the impact of the COVID-19 pandemic on the mortality rate after hip and knee joint arthroplasty.

## 2. Materials and Methods

The present study was performed in accordance with the guidelines of Cochrane Reviews and the Preferred Reporting Items for Systematic Review and Meta-Analysis Protocols guidelines [7,8]. The review protocol was registered with the International Prospective Register of Systematic Reviews (PROSPERO–CRD42022335471). Although the present study involved human participants, ethical approval or informed consent from the participants was not required, because all data were based on previously published studies that were analyzed anonymously without any potential harm to the participants.

### 2.1. Literature Search

We searched the MEDLINE, Embase, and Cochrane Library databases for studies that compared the mortality rates after hip and knee joint arthroplasty surgeries during the COVID-19 pandemic versus pre-pandemic periods. An electronic literature search was performed on March 28, 2022. Following the PICO framework, we defined the following: (1) Patients/population: patients who underwent hip or knee arthroplasty surgery. (2) Intervention: COVID-19 pandemic period. (3) Comparator (control group): pre-pandemic period. (4) Outcome: mortality. The search terms used in the title, abstract, and keyword fields were synonyms and terms related to arthroplasty, COVID-19, and mortality. No language restrictions were applied. The literature search algorithm (including search terms) and results from each database are summarized in Appendix A1. Relevant articles and bibliographies were manually reviewed after the initial online search.

### 2.2. Study Selection

From the titles and abstracts of the studies, two board-certified orthopedic surgeons (K.C.H. and L.E.J.) who worked as faculty in academic centers independently selected the studies for a full-text review. The full text of the article was reviewed if the abstract did not contain sufficient data. We included studies that directly compared the groups that underwent arthroplasty surgeries during the COVID-19 pandemic period and those that underwent surgery during the non-pandemic period (double-arm study), as well as the studies that investigated hip or knee arthroplasty surgeries for any other reasons. We included only original research articles and excluded biomechanical and cadaveric studies, technical notes, letters to the editor, expert opinions, review articles, meta-analyses, conference abstracts, and case reports.

Among studies conducted at identical institutions or those showing similar data during the same follow-up period, the most recent study was included. The κ-value was calculated to determine the inter-reviewer agreement at each stage of study selection. Agreement between the reviewers was correlated a priori with κ values as follows: κ = 1 corresponded to a “perfect” agreement; 1.0 > κ ≥ 0.8 to an “almost perfect” agreement; 0.8 > κ ≥ 0.6 to a “substantial” agreement; 0.6 > κ ≥ 0.4 to a “moderate” agreement; 0.4 > κ ≥ 0.2 to a “fair” agreement; and κ < 0.2 to a “slight” agreement. Disagreements at each stage were resolved via consensus between the two investigators. If consensus could not be reached, it was resolved through discussion with a third investigator, who was also a board-certified orthopedic surgeon.

### 2.3. Data Extraction

To synthesize the qualitative data, the following information and variables were extracted using a standardized form: (1) study design; (2) country in which the study was investigated; (3) number of patients; (4) mean patient age; (4) pandemic study period; (5) type of arthroplasty included—hip/knee; (6) urgency of procedure, (7) screening protocol for COVID-19; (8) number of patients positive for COVID-19; and (9) number of deaths from COVID-19.

For the meta-analysis, the overall mortality rate for any reason (death due to COVID-19-related complications and death unrelated to COVID-19) was investigated as the primary outcome.

Subgroup analyses were performed for (1) mortality within 30-days, (2) mortality unrelated to COVID-19, and (3) mortality after excluding studies where only elective arthroplasties were performed.

As a secondary outcome, the 30-day readmission rate was investigated; additionally, we also compared the mortality rate during the COVID-19 pandemic period among the countries that were investigated.

The predictor variables were the COVID-19 pandemic period and elective/urgent arthroplasty. The outcome variables were mortality (death due to any reason, death due to COVID-19 related complications, and death unrelated to COVID-19) and readmission rate.

For data extraction, the same two board-certified orthopedic surgeons who participated in the study selection independently recorded data from each enrolled study. Disagreements between the reviewers were resolved through discussions between the two investigators.

### 2.4. Risk of Bias Assessment

The methodological quality of the included studies was assessed using the Methodological Index for Nonrandomized Studies (MINORS) [9], which is a valid tool for assessing the quality of both randomized controlled trials and non-randomized studies. The maximum MINORS checklist score for comparative studies was 24. Two independent reviewers performed the quality assessments and resolved disagreements through discussion.

### 2.5. Data Synthesis and Statistical Analyses

The primary outcome of the meta-analysis was the mortality rate following hip or knee arthroplasty in the pandemic and pre-pandemic periods, and the secondary outcome was the readmission rate. For all comparisons, odds ratios (ORs) and 95% confidence intervals (CIs) were calculated as dichotomous data, whereas continuous data were analyzed using the mean difference with 95% CIs. Heterogeneity was assessed using the I^2^ statistic with the Higgins test, in which 25%, 50%, and 75% were considered as low, moderate, and high heterogeneities, respectively. Additionally, the *p*-value was obtained by comparing the statistic with a χ^2^ distribution [10]. Forest plots were used to show the outcomes of the meta-analysis, the pooled estimates of effects, and the overall summary effects of each study that displayed the results from similar individual studies. The statistical significance was set at *p* < 0.05. All data were pooled using a random-effects model, which is recommended to avoid an overestimation of study results, especially in the field of medicine [11]. The fixed-effects model begins with the assumption that the true effect size is similar in all the included studies; thus, we believed that the random-effects model was generally a more plausible match for the current study. We did not perform a test for publication bias because the evaluation of publication bias is recommended only when at least 10 studies are included in the meta-analysis [8]. Statistical analyses were performed using Review Manager (RevMan) software (version 5.3; Copenhagen, Demark) (Nordic Cochrane Center, Cochrane Collaboration 2014) and the “Metafor” package in R (version 3.4.3; R Foundation for Statistical Computing, Vienna, Austria).

## 3. Results

### 3.1. Study Identification

**Figure 1:**
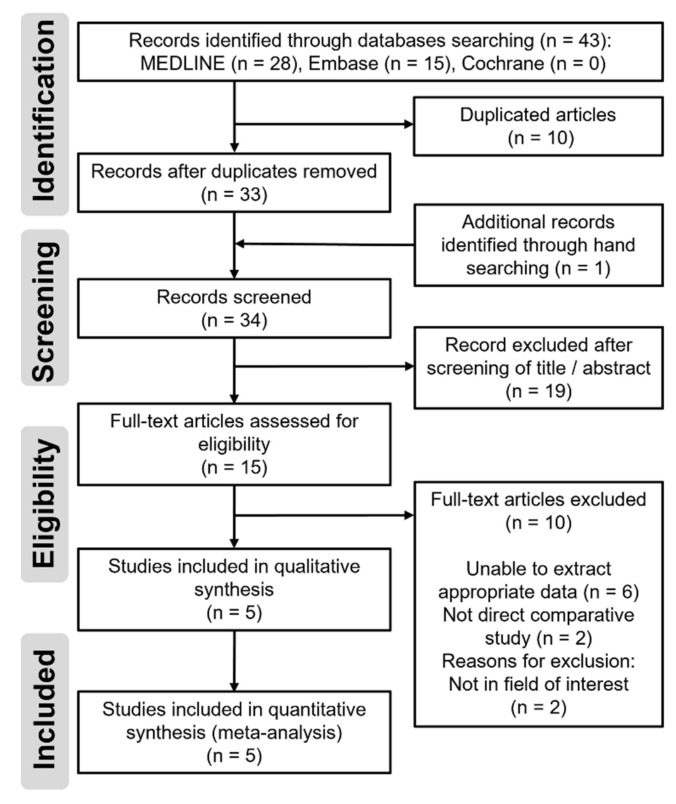
Preferred Reporting Items for Systematic reviews and Meta-Analyses (PRISMA) flow diagram for the process of identifying and selecting the studies included in this meta-analysis.

The process followed for study identification and selection is summarized in Figure 1. The initial electronic literature search yielded 43 articles. After eliminating 10 duplicates and adding one study that was obtained after a manual search, 34 studies were initially screened. Subsequently, 19 studies were excluded after their titles and abstracts were reviewed, and an additional 10 studies were excluded after a full-text review (Table 1). Therefore, the remaining five studies were eligible for data extraction and meta-analysis. The agreement between the reviewers on study selection was “substantial” in the title/abstract review stage (κ = 0.768) and “almost perfect” in the full-text review stage (κ = 0.857).

### 3.2. Study Characteristics and Demographics

Owing to the characteristics of the study design, which compared the pandemic and pre-pandemic periods, all five studies included in the current meta-analysis were retrospective in nature. A total of 3044 patients who underwent hip or knee arthroplasty during the pandemic or pre-pandemic period were included, of which 838 patients underwent arthroplasty during the COVID-19 pandemic period and 2206 patients underwent arthroplasty in the pre-pandemic period. Three studies were conducted in the United Kingdom (UK) [5,12,13]; one study was conducted in Italy [2]; and another study was conducted in India [6]. The mean age was 65.8–78 years in the COVID-19 pandemic group and 64.2–4.6 years in the pre-COVID-19 group. The study period was the first wave of the pandemic in four studies [2,5,6,12] and the second wave in one study [13]. All five studies included both hip and knee arthroplasty in their cohort. The additional details of each study are presented in Table 2.

Four studies included both elective and urgent surgeries [2,5,6,12], and one study only included elective arthroplasties [13]. In three studies, screening for COVID-19 was only performed in symptomatic patients in the early period [2,5,12]. Subsequently, all patients were screened during the late period. In two studies, all the included patients were screened for COVID-19 before admission [6,13]. All the included studies had patients who contracted COVID-19. Three studies reported deaths related to COVID-19 infection [2,5,12]. The additional details of each study are presented in Table 3.

### 3.3. Risk of Bias Assessment

The mean MINORS score for methodological quality assessment was 15.4/24 (range: 15–16) (Table 3). Among the eight main evaluation parameters, all the studies received a point deduction owing to the retrospective study design, the lack of prospective calculation of sample size, and the lack of unbiased assessment of the study endpoint. Four studies received a point deduction because the follow-up period was insufficient or because they did not describe the length of the follow-up [2,6,12,13]. In the additional criteria, all studies received a point deduction because they performed a historical comparison. The MINORS score indicated that the control and study groups should be managed during the same period [9]. Three studies received a point deduction because their baseline characteristics of patients were different between the pandemic and pre-pandemic periods in that the proportion of urgent procedures increased during the pandemic period [5,6,12].

### 3.4. Meta-Analysis

#### 3.4.1. Overall Mortality Rate

**Figure 2:**
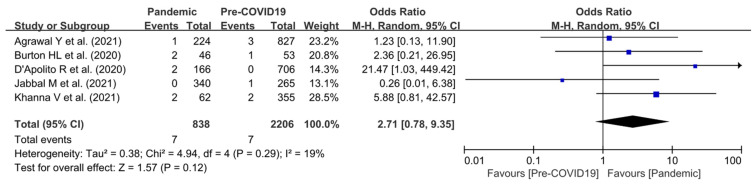
Forest plot comparing overall mortality of patients following hip or knee arthroplasty between the pandemic and pre-pandemic periods.

We extracted data of overall mortality following hip or knee arthroplasty from the group that underwent arthroplasty during the pandemic period (838 patients) and the group that underwent arthroplasty during the pre-pandemic period (2206 patients). The overall mortality rate was 7/838 during the pandemic period and 7/2206 in the pre-pandemic period for mortality due to any reason (both “related to COVID-19” and “unrelated to COVID-19”) and showed no significant differences between the pandemic and pre-pandemic periods (OR, 2.71; 95% CI, 0.78–9.35; *p* = 0.12). The heterogeneity considered low (I^2^ = 19%). Additional forest-plot details are shown in Figure 2.

#### 3.4.2. Subgroup Analyses for Mortality

Four studies [5,6,12,13] addressed mortality within 30 days following hip or knee arthroplasty during both the pandemic and pre-pandemic periods. There were also no significant differences in the mortality following lower-limb arthroplasties within 30 days (OR, 2.28; 95% CI, 0.69–7.57; *p* = 0.18). The heterogeneity considered low (I^2^ = 0%).

We extracted the data of mortality unrelated to COVID-19 (OR, 1.59; 95% CI, 0.40–6.37; *p* = 0.51; I^2^ = 10%). Four patients died due to COVID-19 in five of the included studies. Thus, the mortality rate unrelated to COVID-19 was 3/838 patients during the pandemic period and 7/2206 patients in the pre-pandemic period and showed no significant differences between the pandemic and pre-pandemic periods (OR, 1.41; 95% CI, 0.33–6.07; *p* = 0.64). The heterogeneity was considered low (I^2^ = 18%).

**Figure 3:**
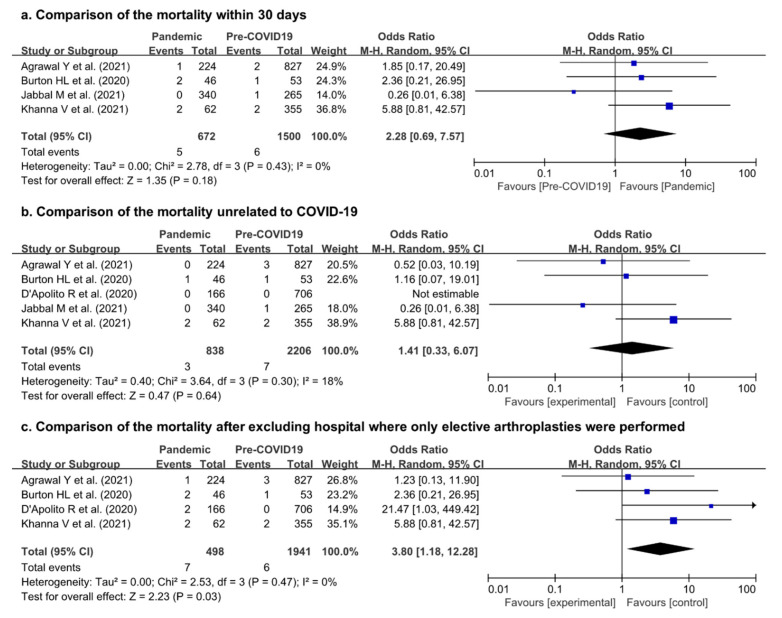
Forest plot comparing the mortality of patients following hip or knee arthroplasty (**a**) within 30 days, (**b**) unrelated to COVID-19, and (**c**) after excluding hospitals where only elective arthroplasties were performed between the pandemic and pre-pandemic periods [2,5,6,12,13].

After excluding one study that only included elective surgeries [13], the mortality rate was compared with that of four studies that included both elective and urgent arthroplasties [2,5,6,12]. The mortality was significantly higher in the pandemic period than in the pre-pandemic period (OR, 3.80; 95% CI, 1.18–12.28; *p* = 0.03). The heterogeneity was considered low (I^2^ = 0%). Additional forest-plot details are shown in Figure 3.

#### 3.4.3. Readmission Rate within 30 Days

**Figure 4:**
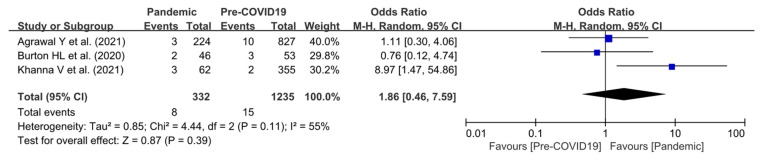
Forest plot comparing the readmission rate within 30 days following hip or knee arthroplasty between the pandemic and pre-pandemic periods [5,6,12].

Three studies addressed readmission within 30 days following hip or knee arthroplasty in both the COVID-19 pandemic and pre-pandemic periods [5,6,12]. The readmission rate did not present significant differences between the pandemic and pre-pandemic periods (OR, 1.86; 95% CI, 0.46–7.59; *p* = 0.39). The heterogeneity was considered moderate (I^2^ = 55%). Additional forest-plot details are shown in Figure 4.

#### 3.4.4. Comparison of Mortality Rate Following Hip and Knee Joint Arthroplasty in Each Country

**Figure 5:**
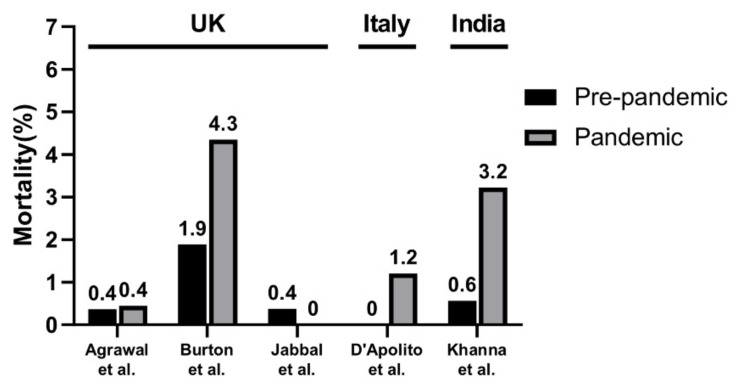
Visualization of overall mortality in included studies.

In the included studies, data of the mortality rates in the UK, Italy, and India were provided. Mortality was reported to be from 0% to 4.35% in the UK, 1.20% in Italy, and 3.23% in India during the pandemic period. In the pre-pandemic period, in the included studies, mortality was reported to be from 0.4% to 1.9% in the UK, 0% in Italy, and 0.6% in India (Figure 5).

## 4. Discussion

In the present study, the mortality rate during the COVID-19 pandemic period following hip and knee arthroplasty surgeries did not show a significant increase. In a subgroup analysis of hospitals where patients underwent urgent arthroplasties as well as elective arthroplasties, the pandemic period presented a higher mortality rate than the pre-pandemic period. Thus, we believe that arthroplasty can be safely performed as elective surgery without additional risks. Caution should be exercised when urgent arthroplasties are performed during a pandemic period.

With a high fatality rate for COVID-19, non-urgent surgeries were likely to be delayed during the early first wave (March 2020), regardless of COVID-19 contraction. Additionally, there were no concrete guidelines for elective surgery during the pandemic period. Regarding the first wave of the COVID-19 pandemic, there are studies that evaluated the impact of the pandemic on the mortality of patients undergoing elective orthopedic surgery. Stevenson et al. presented 1% 30-day mortality following orthopedic oncology surgery during the pandemic and concluded that major orthopedic surgery procedures could continue without significant concerns [14]. In the present study, although we could not extract separate data for elective surgery, the pandemic period did not demonstrate significant increases in overall mortality and readmission rates. We assume that a low proportion of positive tests for COVID-19 (12/838 (1.4%)) and the well-controlled health status of patients undergoing elective arthroplasty contributed to the results. We believe that elective arthroplasty can be safely performed, with thorough preparation and close monitoring.

In contrast, for urgent arthroplasty, COVID-19 could not be fully detected in asymptomatic patients during the incubation period. In addition, hospital admission for traumatic hip fracture was prone to delay during the COVID-19 pandemic [15]; the preoperative morbidity of injured patients was likely to be uncontrolled. These factors put patients more at risk, even though they were negative for COVID-19, and lockdown could aggravate these factors. In the present study, there was a significant increase in the mortality rate in the subgroup analysis for hospitals where urgent arthroplasties, as well as elective procedures, were performed. Three studies described that they did not screen all patients in the early period of the first wave but tested symptomatic patients. In addition, the clinical situation of urgent arthroplasty is likely to underestimate the symptoms of COVID-19. In a report from New York City, there was a marked increase in the mortality of patients who contracted to COVID-19 following hip fracture of up to 35.3% [16]. Because Dar et al. reported that delayed surgical intervention did not affect the early postoperative mortality rate [17], it would be safe to repeatedly check for COVID-19 and observe closely, with surgical delay for a few days, if there are pulmonary symptoms in patients who have to undergo urgent arthroplasty.

A systematic review of the mortality rate of hip fracture surgery between the pre- and COVID-19 pandemic periods was conducted by Tripathy et al. [18]. In their analysis, no significant differences were observed in 30-day mortality following hip fracture. In contrast, increased mortality was demonstrated in the subgroup analysis in the present study after excluding hospitals where only elective arthroplasties were performed, resulting in a relatively high proportion of urgent surgeries. There are several reasons for this finding. First, they included cases of conservative treatment, which could have influenced the results, with relatively higher mortality [19]. Second, the study differs from the present study with respect to the disease group and surgical type, in that they only targeted hip fractures and included open reduction and internal fixation. Thus, the studies included in the two meta-analyses were completely different and did not show any overlap. Third, the two studies included in their analysis did not have any COVID-19-positive patient; however, all studies in our analysis had at least one COVID-19-positive patient. Thus, we believe that the present study could better reflect the pandemic period and had its own strengths.

Nearly 2 years after the COVID-19 pandemic, the Omicron variant was the most dominant variant. During Omicron, five- to eight-fold increased transmissibility and three-fold reduced mortality were reported compared with previous periods, characterized by variants such as the Gamma and Delta variants [20]. Because our data were based on the first and second waves of COVID-19, which showed >30% mortality in patients with COVID-19 and concomitant hip fracture [16], the application of the results of the present study to the recent Omicron period is limited. Instead, for a future situation of a pandemic with high morbidity and mortality, the data of the present study can have implications for both elective and urgent/emergent arthroplasties.

The present study had some limitations. First, elective and urgent arthroplasties were not completely differentiated, although we performed a subgroup analysis for hospitals that included urgent arthroplasties. Second, cases of hip and knee arthroplasties were mixed. Because the cause and mortality for each type of arthroplasty were quite different, the present study had a limitation in this aspect with characteristics for meta-analysis. Third, the proportions of elective and urgent arthroplasties were different during the pre-pandemic and COVID-19 pandemic periods. Because elective arthroplasty was reduced during the pandemic period, especially during lockdown, the proportion of urgent arthroplasty increased, which could have interfered with the mortality rate in the present study. Fourth, there were a relatively small number of patients in this study who died; thus, the statistical findings were weak.

Despite these several limitations, nowadays, the COVID-19 pandemic still presents important issues in the clinical field. We believe the results of the current study might be meaningful, especially regarding patients who underwent hip or knee arthroplasties in this pandemic.

## 5. Conclusions

Based on our meta-analysis, the overall mortality rate in the COVID-19 pandemic period following hip and knee arthroplasty did not show a significant increase. This finding could help maintain the practice of elective arthroplasty in a future pandemic situation.

## Figures and Tables

**Table 1:** Studies that were excluded at full-text review and reasons for exclusion.

	Comparison	Reason for Exclusion
De C. et al. (2021) [7]	Mortality after hip fracture (pandemic vs. pre-pandemic)	Impossible to extract the data of arthroplasty
Konda, S.R. et al. (2020) [3]	Mortality after hip fractures (COVID-19 infection vs. control)	Impossible to extract the data of arthroplasty
Malik-Tabassum, K. et al. (2021) [8]	Mortality after hip fracture (pandemic vs. pre-pandemic)	Impossible to extract the data of arthroplasty
Meena, O.P. et al. (2021) [9]	Mortality after arthroplasty for arthritis during pandemic period	No comparison between pandemic and pre-pandemic periods (single-arm study)
Slullitel, P.A. et al. (2020) [12]	Mortality after hip fracture (pandemic vs. pre-pandemic)	Impossible to extract the data of arthroplasty
Stoneham, A.C.S. et al. (2020) [4]	Mortality after arthroplasty for trauma during pandemic period	No comparison between pandemic and pre-pandemic periods (single-arm study)
Thakrar, A. et al. (2020) [13]	Mortality after hip fracture (pandemic vs. pre-pandemic)	Impossible to extract the data of arthroplasty
Wignall, A. et al. (2021) [10]	Mortality after hip fracture (pandemic vs. pre-pandemic)	Impossible to extract the data of arthroplasty

**Table 2:** Study design, demographic data, and study details of the included studies.

Author (Year)	Study Design	Country	Sample Size		Mean Age		Pandemic Study Period	Type of Arthroplasty
			P	Pre-P	P	Pre-P		
Agrawal, Y. et al. (2021) [5]	RCS	UK	224	827	69.4 (35 to 91)	66.8 (17 to 96)	Mar/01/2020–May/31/2020	Hip, knee
Burton, H.L. et al. (2020) [12]	RCS	UK	46	53	78 (58 to 108)	74.6 (45 to 88)	Apr/01/2020–Jun/16/2020	Hip, knee
D’Apolito, R. et al. (2020) [2]	RCS	Italy	166	706	N/A	N/A	Feb//24/2020–Apr/10/2020	Hip, knee
Jabbal, M. et al. (2021) [13]	RCS	UK	340	265	68.5 (28 to 90)	N/A	Jul/01/2020–Jan/31/2021	Hip, knee
Khanna, V. et al. (2021) [6]	RCS	India	62	355	65.77 ± 12.26	64.23 ± 10.98	Mar/01/2020–Aug/31/2020	Hip, knee

Abbreviations: P, pandemic; Pre-P, pre-pandemic; N/A, not available; RCS, retrospective cohort study; UK, United Kingdom.

**Table 3:** Urgency of procedure, details regarding COVID-19, and MINORS score.

Author (Year)	Urgency of Procedure		COVID-19 Screening	COVID-19 Positive	Death of COVID-19 Patients	MINORS Score
	P	Pre-P				
Agrawal, Y. et al. (2021) [5]	Elective (167) Urgent (57)	Elective (802) Urgent (24)	Early period: symptomatic patients Late period: all patients	6/47	1	16
Burton, H.L. et al. (2020) [12]	Elective (2) Urgent (44)	Elective (36) Urgent (17)	Early period: symptomatic patients Late period: all patients	1/41	1	15
D’Apolito, R. et al. (2020) [2]	Elective (159) Urgent (7)	Elective (625) Urgent (31)	Early period: symptomatic patients Late period: all patients	2/NA	2	15
Jabbal, M. et al. (2021) [13]	Elective (340)	Elective (265)	All patients	1/340	0	16
Khanna, V. et al. (2021) [6]	Elective and urgent (62)	Elective and urgent (355)	All patients	2/62	0	15

Abbreviations: P, pandemic; Pre-P, pre-pandemic; MINORS, Methodological Index for Non-randomized Studies.

## Data Availability

Not applicable.

## Appendix A

**Table A1:** Literature search algorithm and results from relevant clinical studies.

	**PubMed (19 March 2022).**	
	**Search Queries**	**Number of Articles**
#1	“arthroplast * ” (Title/Abstract)	77,820
#2	“arthroplasty” (MeSH Terms) OR “hemiarthroplasty” (MeSH Terms)	80,950
#3	Search (#1 OR #2)	107,943
#4	“COVID” (Title/Abstract) OR “SARS-COV-2” (Title/Abstract)	223,760
#5	“COVID-19” (MeSH Terms) OR “SARS-COV-2” (MeSH Terms)	148,502
#6	Search (#4 OR #5)	233,472
#7	Search (#3 AND #6)	216
#8	“mortalit *” (Title/Abstract)	893,827
#9	“mortality” (MeSH Terms)	416,458
#10	Search (#8 OR #9)	1,149,574
#11	Search (#7 AND #10)	28
	**Embase (19 March 2022).**	
	**Search Queries**	**Number of Articles**
#1	arthroplast *:ti,ab,kw	91,951
#2	hemiarthroplast *:ti,ab,kw	4517
#3	Search (#1 OR #2)	93,979
#4	covid *:ti,ab,kw	221,739
#5	COVID-19:ti,ab,kw	217,672
#6	SARS-COV-2:ti,ab,kw	81,114
#7	Search (#4 OR #5 OR #6)	237,133
#8	mortalit *:ti,ab,kw	1,315,660
#9	Search (#3 AND #7 AND #8)	15
	**Cochrane Library (19 March 2022).**	
	**Search Queries**	**Number of Articles**
#1	arthroplast *:ti,ab,kw	13,388
#2	hemiarthroplast *:ti,ab,kw	529
#3	Search (#1 OR #2)	13,611
#4	covid *:ti,ab,kw	9797
#5	COVID-19:ti,ab,kw	9308
#6	SARS-COV-2:ti,ab,kw	3690
#7	Search (#4 OR #5 OR #6)	9995
#8	mortalit *:ti,ab,kw	94,978
#9	Search (#3 AND #7 AND #8)	0

## References

[1] Paul K.D., Levitt E., McGwin G., Brabston E.W., Gilbert S.R., Ponce B.A., Momaya A.M. (2021). COVID-19 Impact on Orthopedic Surgeons: Elective Procedures, Telehealth, and Income. South. Med. J..

[2] D’Apolito R., Faraldi M., Ottaiano I., Zagra L. (2020). Disruption of Arthroplasty Practice in an Orthopedic Center in Northern Italy During the Coronavirus Disease 2019 Pandemic. J. Arthroplast..

[3] Forlenza E.M., Higgins J.D., Burnett R.A., Serino J., Della Valle C.J. (2022). COVID-19 Infection After Total Joint Arthroplasty Is Associated With Increased Complications. J. Arthroplast..

[4] Stoneham A.C.S., Apostolides M., Bennett P.M., Hillier-Smith R., Witek A.J., Goodier H., Asp R. (2020). Early outcomes of patients undergoing total hip arthroplasty for trauma during COVID-19. Bone Jt. Open.

[5] Agrawal Y., Vasudev A., Sharma A., Cooper G., Stevenson J., Parry M.C., Dunlop D. (2021). Morbidity and mortality in patients undergoing lower limb arthroplasty surgery during the initial surge of the COVID-19 pandemic in the UK at a single-speciality orthopaedic hospital. Bone Jt. Open.

[6] Khanna V., Nashikkar P.S., Mahajan R., Tripathi S. (2021). Impact of Covid-19 pandemic on arthroplasty services and early experience after resuming surgeries at a ‘non Covid’ center. J. Clin. Orthop. Trauma.

[7] Moher D., Shamseer L., Clarke M., Ghersi D., Liberati A., Petticrew M., Shekelle P., Stewart L.A., Group P.-P. (2015). Preferred reporting items for systematic review and meta-analysis protocols (PRISMA-P) 2015 statement. Syst. Rev..

[8] Higgins J.P., Thomas J., Chandler J., Cumpston M., Li T., Page M.J., Welch V.A. (2019). Cochrane Handbook for Systematic Reviews of Interventions.

[9] Slim K., Nini E., Forestier D., Kwiatkowski F., Panis Y., Chipponi J. (2003). Methodological index for non-randomized studies (minors): Development and validation of a new instrument. ANZ J. Surg..

[10] Higgins J.P., Thompson S.G., Deeks J.J., Altman D.G. (2003). Measuring inconsistency in meta-analyses. BMJ.

[11] Schmidt F.L., Oh I.S., Hayes T.L. (2009). Fixed- versus random-effects models in meta-analysis: Model properties and an empirical comparison of differences in results. Br. J. Math. Stat. Psychol..

[12] Burton H.L., Burden E., King A., Kassam A.A., Hubble M.J., Toms A.D. (2020). Urgent Arthroplasty Interventions During the COVID-19 Pandemic: Operating Risks in Low-Prevalence Areas. Cureus.

[13] Jabbal M., Campbel N., Savaridas T., Raza A. (2021). Careful return to elective orthopaedic surgery in an acute hospital during the COVID-19 pandemic shows no increase in morbidity or mortality. Bone Jt. Open.

[14] Stevenson J.D., Evans S., Morris G., Tillman R., Abudu A., Jeys L., Parry M. (2020). Mortality of high-risk orthopaedic oncology patients during the COVID-19 pandemic: A prospective cohort study. J. Surg. Oncol..

[15] Jarvis S., Salottolo K., Madayag R., Pekarek J., Nwafo N., Wessel A., Duane T., Roberts Z., Lieser M., Corrigan C. (2021). Delayed hospital admission for traumatic hip fractures during the COVID-19 pandemic. J. Orthop. Surg. Res..

[16] Egol K.A., Konda S.R., Bird M.L., Dedhia N., Landes E.K., Ranson R.A., Solasz S.J., Aggarwal V.K., Bosco J.A., Furgiuele D.L. (2020). Increased Mortality and Major Complications in Hip Fracture Care During the COVID-19 Pandemic: A New York City Perspective. J. Orthop. Trauma.

[17] Dar G.N., Wani M.I., Mumtaz M.U., Kawoosa A.A., Ali N. (2021). Delayed Fixation of Hip Fractures and Short-term Outcome in Coronavirus Positive Patients: A Prospective Cohort Study. J. Clin. Diagn. Res..

[18] Tripathy S.K., Varghese P., Panigrahi S., Panda B.B., Velagada S., Sahoo S.S., Naik M.A., Rao S.K. (2021). Thirty-day mortality of patients with hip fracture during COVID-19 pandemic and pre-pandemic periods: A systematic review and meta-analysis. World J. Orthop..

[19] Tay E. (2016). Hip fractures in the elderly: Operative versus nonoperative management. Singap. Med. J..

[20] Ribeiro Xavier C., Sachetto Oliveira R., da Fonseca Vieira V., Lobosco M., Weber dos Santos R. (2022). Characterisation of Omicron Variant during COVID-19 Pandemic and the Impact of Vaccination, Transmission Rate, Mortality, and Reinfection in South Africa, Germany, and Brazil. BioTech.

